# On Chlorotic Affections

**Published:** 1800-03

**Authors:** 


### *On Chlorotic Affections; communicated by* Dr. Scott.

[ Continued from our laſt Number, p. 108. ]

"IN this reſpect, ſhe feels entirely dependent upon others; and that in a great degree it does not reſt with herſelf, whether ſhe is even to taſte the ſweets of matrimony, or to lead an inſulated life; without a tender friend to be her confidant and ſupporter, and without the dear pledges of ſuch a friendſhip, to be the ſolace of her declining years. No wonder, therefore, that her full boſom ſhould ſwell ſo much with hopes and fears, occaſioning that languid and deſponding ſtate of mind, in which the ſympathizing body ſoon participates.

"Young men, of about the ſame age, who have been prevented by circumſtances from aſſociating ſo freely with their equals in years as they ſhould do, who have been kept under an improper conſtraint, or who are as yet undetermined about, and are daily brooding over, what particular occupation of life they are to follow, eſpecially if they poſſeſs a warm fancy and keen feelings, are ſometimes affected with what we may juſtly call *chloroſis*; they become wan and emaciated, deſpondent and indolent: averſe from both bodily and mental exertion, and wanting reſolution to encounter the ſtorms of active life, they court ſolitude, and indulge in the reveries of their own imagination. Moſt commonly ſhame, and a returning ſenſe of the propriety and neceſſity of their taking a part in the multifarious duties of the buſy world, and the ſolicitation of friends, put a ſtop to this affection, by calling off the mind from its own feelings. Although, I believe, that this ſtate ſometimes goes on increaſing, or continues ſo long as to lay the foundation for, or to bring into action, a prediſpoſition to other more dangerous and fatal diſeaſes, as conſumption of the lungs, &c. we have well authenticated inſtances on record, of men having even fallen a ſacrifice to miſtaken notions of religion, in denying themſelves the enjoyment of the ſex. One poet, Michael Verini, of Florence, has very feelingly deſcribed (ſee Roſcoe's Life of Lorenzo de Medici, Vol. II. Appendix) his own ſenſations in this ſituation, and ſeems to have been perfectly aware of the fate he had to expect, by perſiſting in his rigid continence.

"If our hypotheſis be well founded, we ſhould very ſeldom meet with the diſeaſe in the lower claſs of females, or at leaſt much leſs frequently than in the middling and higher claſſes, as their condition puts them in ſome meaſure on the ſame footing with the male; the cares of life, and the procuring the means means of fubfiftence, engroffing moft commonly their whole attention; while they neither are under the fame reftraints, nor have received the fame ideas of female honour, as thofe above them. The difeafe too, fhould be almoft unknown in favage life; and its occurrence fhould be connected with the progreffion of fociety, and of refinement of manners. It would therefore be neceffary to inquire, whether it occurs oftener in fome countries than in others? whether it was equally common in ancient as in modern times? whether the peculiar cuftoms of different countries, or particular modes of education, affect its frequency? and whether it is as often met with in the reclufe and country, as in the buftling and city life? It would be likewife requifite to compare its fymptoms, &c. with thofe of difeafes, which, according to obfervation, derive their origin from, or are fomehow connected with particular paffions and emotions of mind. We fhould inquire too, whether (as is fuppofed in what would be then its congener, *noftalgia*) a peculiarity in the fcenery and general habitude of the place of nativity or refidence, influenced its occurrence, by working on the imagination, and giving a turn to the fancy and general affociation of ideas?

"I am well aware that this hypothefis will be found open to many objections, and that it is far from giving us a fatisfactory explanation of the difeafe. I only fuggeft the neceffity of attending to the fubject, as from it there may perhaps arife, to a philofophical inquirer, the means of averting, in fome meafure, a malady which fo often preys upon blooming beauty, by a proper direction of the paffions during education, and in early life; by calling off the mind, at this important crifis, from its own anxious and painful furmifes, and by engaging the attention in fome active occupation. I would, however, beg leave to remark, that in the difcuffion of this queftion, extreme delicacy and caution fhould be obferved, left the deceitful fophifter fhould therein find a pretence to impofe on us his iniquitous pretended improvements; or the artful libertine, a lure to enfnare the unfufpicious and unguarded female in the mazes of vice."

